# Association of atypical anti-neutrophil cytoplasmic antibody with comorbidities and outcome in a hospital-based population

**DOI:** 10.1016/j.heliyon.2024.e24105

**Published:** 2024-01-06

**Authors:** Chiao-Chi Ou, Yen-Ching Wu, Jun-Peng Chen, Wen-Nan Huang, Yi-Hsing Chen, Yi-Ming Chen

**Affiliations:** a Division of Allergy, Immunology, and Rheumatology, Department of Internal Medicine, Taichung Veterans General Hospital, Taichung, Taiwan; b Department of Industrial Engineering and Enterprise Information, Tunghai University, Taichung, Taiwan; c Department of Medical Research, Taichung Veterans General Hospital, Taichung, Taiwan; d School of Medicine, College of Medicine, National Yang Ming Chiao Tung University, Taipei, Taiwan; e College of Business and Management, Ling Tung University, Taichung, Taiwan; f Department of Post-Baccalaureate Medicine, College of Medicine, National Chung Hsing University, Taichung, Taiwan; g Institute of Biomedical Science and Rong Hsing Research Center for Translational Medicine, National Chung Hsing University, Taichung, Taiwan; h Precision Medicine Research Center, College of Medicine, National Chung Hsing University, Taichung, Taiwan

**Keywords:** Anti-neutrophil cytoplasmic antibody, Comorbidity, Kidney survival, Mortality

## Abstract

**Introduction:**

Atypical anti-neutrophil cytoplasmic antibody (a-ANCA) is characterized by a positive fluorescence staining other than typical cytoplasmic or perinuclear ANCA. ANCA is associated with increased risk of dialysis and mortality in patients with ANCA vasculitis. However, comorbidities related to a-ANCA and whether a-ANCA exhibits an increased risk for renal failure and mortality remain unclear. This study aimed to explore the comorbidities and outcome associated with a-ANCA.

**Materials and methods:**

This retrospective study enrolled 164 and 170 patients with typical ANCA and a-ANCA positivity, respectively, who visited Taichung Veterans General Hospital, Taiwan from January 2016 to March 2021. Logistic regression analysis was used to determine risk factors and the rheumatological diagnosis associated with a-ANCA. Cox proportional hazard regression and Kaplan–Meier curves were employed to identify variables associated with 5-year renal survival and mortality.

**Results:**

Patients with a-ANCA had lower chance of ANCA-associated vasculitis (OR: 0.02, 95 % CI: 0.01–0.07 *p* < 0.001), and systemic lupus erythematosus (OR: 0.23, 95 % CI: 0.11–0.48, *p* < 0.001), but a higher risk of rheumatoid arthritis (OR: 2.99, 95 % CI: 1.15–7.83, *p* = 0.025) and ulcerative colitis (OR: 5.50, 95 % CI: 1.20–25.29, *p* = 0.028). Patients with a-ANCA had a better renal survival (OR: 0.14, 95 % CI: 0.08–0.24, *p* < 0.001) and lower mortality (OR: 0.31, 95 % CI: 0.16–0.60, *p* = 0.001) than patents in the typical ANCA group. The 5-year renal survival and mortality was 89.3 % and 8.8 %, respectively, in patients with a-ANCA.

**Conclusion:**

Patients with a-ANCA had better renal survival and lower mortality rates compared to patients with typical ANCA. These real-world data provide evidence of the long-term outcome and shed light on avenues for the strategic management of patients with a-ANCA.

## 1 Introduction

Anti-neutrophil cytoplasmic antibody (ANCA) was first identified in 1982 by Davies et al. using indirect immunofluorescence to detect the presence of ANCAs [1,2]. This discovery was subsequently detailed in patients with ANCA-associated vasculitis (AAV) [2]. Atypical ANCA (a-ANCA) was described in 1990 as presenting a faint diffuse and homogenous cytoplasmic staining in addition to a strong linear perinuclear staining [3,4]. This staining pattern is entirely different from the typical diffuse and granular cytoplasmic fluorescence of ANCA [3]. The antigen of a-ANCA remains unclear although bactericidal/permeability-increasing protein, catalase, cathepsin G, enolase, or lactoferrin, have been implicated as putative target antigens [5]. A-ANCA are present in patients with inflammatory bowel disease (IBD), including ulcerative colitis (UC) and Crohn's disease (CD), primary sclerosing cholangitis (PSC), autoimmune hepatitis (AIH), and AIH/PSC overlap syndrome [[5], [6], [7]]. The sensitivity of atypical *p*-ANCA is 37–83 % in patients with UC [[8], [9], [10], [11], [12], [13]], 10–25 % in those with CD [[8], [9], [10], [11], [12], [13]], 65–95 % in those with PSC [8,14], and 36–83 % in those with AIH [9,14,15], respectively. However, no study has comprehensively investigated the association of a-ANCA and various systemic autoimmune diseases.

The association between AAV and c-ANCA (proteinase 3, PR3), *p*-ANCA (myeloperoxidase, MPO) that react with neutrophil cytoplasmic components had been identified [2,[16], [17], [18]]. AAV including granulomatosis with polyangiitis (GPA), microscopic polyangiitis (MPA), and eosinophilic granulomatosis with polyangiitis (Churg–Strauss syndrome, EGPA) [19]. A recent study showed that MPO-ANCA-positive patients had worse renal prognosis than PR3-ANCA-positive patients [20]. Moreover, there had 80 % became renal involvement in AAV patients [21]. In addition, a 2.7-fold increase in the mortality rate was ascertained in patients with AAV compared to that of the general population [22]. A previous report indicated that mortality rates of patients with AAV during follow-up of 1 year is 25.5 % [23]. The prognosis of patients with typical ANCA, including c-ANCA and *p*-ANCA, is well-established. However, there are limited data of the prognostic value of a-ANCA. Therefore, it is unclear whether a-ANCA is associated with renal damage and mortality, especially in comparison with typical ANCA, in the population tested for ANCA.

The primary aim of this study was to examine the correlation between a-ANCA and comorbidities, renal survival, and mortality in a real-world, hospital-based population.

## 2 Materials and methods

### 2.1 Study population

From January 2016 to March 2021, 10,230 patients who visited the outpatient clinic or emergency department or were admitted to the inpatient ward of Taichung Veterans General Hospital, Taiwan and underwent examination for anti-neutrophil cytoplasmic antibody by indirect immunofluorescence (IIF) were enrolled. Inclusion criteria involved having undergone ANCA testing at least once and testing positive for typical ANCA and a-ANCA. We excluded 9886 patients tested negative for ANCA and those presenting with atypical c-ANCA, specifically excluding individuals diagnosed with AIH and PSC to minimize the heterogeneity of the data. In total, 30 and 134 patients with positivity for c-ANCA and *p*-ANCA, and 170 patients with positivity for a-ANCA, respectively were included in the analysis. This study was conducted in accordance with the Declaration of Helsinki and was approved (CE22213A) by the Institutional Review Board of Taichung Veterans General Hospital, Taiwan. As these data were analyzed anonymously, the requirement of informed consent was waived by the approving authority.

### 2.2 Study design and data collection

This retrospective study extracted data from electronic health records. The index date was the date when the ANCA test was performed. The patients were divided into two groups: one consisting of typical ANCA, including c-ANCA and *p*-ANCA, and the other consisting of a-ANCA. The study analyzed and compared age, gender, disease classification, laboratory data, and medication usage between the two groups. ANCA titers, anti-MPO levels, anti-PR3 levels, anti-nuclear antibodies (ANA) results, RBC counts, HGB levels, platelet counts, WBC counts, neutrophil counts, serum creatinine levels, CRP levels, and urine protein/creatinine ratio (UPCR) were collected from the nearest available data interval to the index date. Additionally, the analysis included medications such as glucocorticoids, azathioprine, cyclophosphamide, methotrexate (MTX), and rituximab that were prescribed after the index date.

The diagnosis of AAV were categorized according to 2022 American College of Rheumatology (ACR)/European Alliance of Associations for Rheumatology (EULAR) classification criteria of MPA, GPA and EGPA [[24], [25], [26]]. The classification of SLE was determined by the 2019 EULAR/ACR criteria [27]. Renal replacement therapy included procedure codes for hemodialysis, peritoneal dialysis, or kidney transplant. The other comorbidities were used the International Classification of Diseases, Ninth Revision, Clinical Modification (ICD-9-CM) and ICD-10-CM codes that were recorded twice in the outpatient clinics or once in the inpatient system to identify comorbidities that were present within 6 months prior to the index date (Supplement Table 1).

### 2.3 Autoantibody detection

The identification of a-ANCA is accomplished through an IIF assay on neutrophils, which are detected with FITC-conjugated goat anti-human IgG-secondary antibodies (Inova Diagnostics, CA, USA). This process is characterized by a rim-like staining of the nuclear periphery along with multiple intranuclear fluorescent foci on an ethanol-fixed substrate [28], while yielding negative results on a formalin-fixed substrate. This staining pattern is distinguishable from the typical ANCA pattern, which presents with diffuse and granular cytoplasmic fluorescence staining; a titer ≥1:20 that is reviewed by a senior medical technologist is considered positive. To avoid misclassification of typical ANCA and a-ANCA, immunofluorescence assays were performed on both ethanol- and formalin-fixed substrates to prevent the rearrangement of charged cellular components around the nucleus, which has been reported to reduce the risk of misclassification [29]. ANCA-MPO and ANCA-PR3 are detected using the fluorescence enzyme immunoassay (FEIA; Thermo Fisher Scientific, MA, USA).

### 2.4 Statistical analysis

Data are presented as the number (proportion) for categorical variables and the median (interquartile range) for continuous variables. Categorical variables were compared using the chi-square test, and continuous variables were compared using the Kruskal-Wallis test. Factors associated with a-ANCA were examined using logistic regression analysis, after adjustment for age and sex, and the results are presented as the odds ratio (OR) with the 95 % confidence interval (Cl). Variables associated with renal survival and mortality were analyzed using Cox proportional hazard regression to calculate hazard ratio (HR) with 95 % Cl. The study's sample size of 334 was determined through a power analysis for Cox regression, indicating 87 % power at a 0.050 significance level to detect a regression coefficient of 0.310, affirming its sufficiency for the regression models in the study. The 5-year renal survival and mortality rates was compared between patients with typical ANCA and a-ANCA using the Kaplan–Meier curve. All data were analyzed using SPSS version 22.0 (IBM Corp., Armonk, NY, USA). A *p*-value <0.05 was considered statistically significant.

## 3 Results

### 3.1 General characteristics of the study population

Demographic data for participants with c-ANCA, *p*-ANCA and a-ANCA are shown in Table 1. We enrolled 334 ANCA-positive patients, thus including 30 participants with c-ANCA, 134 with *p*-ANCA and 170 with a-ANCA. The median follow-up time was 2.2 (range, 0.6–4.1) years. We found that in patients with c-ANCA, 10 (33.3 %) had AAV, 1 (3.3 %) had RA, and 10 (33.3 %) had SLE. In patients with *p*-ANCA, 62 (46.3 %) had AAV, 5 (3.7 %) had RA, and 25 (18.7 %) had SLE. In patients with a-ANCA, 3 (1.8 %) had AAV, 17 (10.0 %) had RA, and 10 (5.9 %) had SLE. Compared to patients with c-ANCA, those with a-ANCA were more likely to be older and to have lower anti-PR3 levels. Additionally, ANCA titers, RBC and HGB levels were higher in the a-ANCA group than in the c-ANCA group. Furthermore, AAV and SLE were less frequently reported in patients with a-ANCA than in those with c-ANCA. In contrast, CKD was more frequently reported in the c-ANCA group. Compared to patients with *p*-ANCA, those with a-ANCA were more likely to have lower ANCA titers, anti-MPO levels, and creatinine levels. In addition, RBC and HGB levels were higher in the a-ANCA group than in the *p*-ANCA group. Moreover, hypertension, CKD, AAV, SLE were less frequently reported in patients with a-ANCA than those in the *p*-ANCA group. Interestingly, we discovered that patients with a-ANCA were associated with UC compared to the typical ANCA group.

**Table 1:** Comparisons of baseline characteristics in patients with c-ANCA, *p*-ANCA and atypical ANCA.

	Atypical ANCA (n = 170)		C-ANCA (n = 30)		P-ANCA (n = 134)		*p* value
Age	61.0	(43.0–73.0)	47.0	(31.5–63.5)	62.0	(50.8–75.3)	0.008^†#^
Sex							0.477
Female	99	(58.2 %)	21	(70.0 %)	81	(60.4 %)	
Male	71	(41.8 %)	9	(30.0 %)	53	(39.6 %)	
ANCA titer							<0.001^†‡#^
1:20	70	(41.2 %)	12	(40.0 %)	32	(23.9 %)	
1:40	44	(25.9 %)	10	(33.3 %)	14	(10.4 %)	
≥1:80	56	(32.9 %)	8	(26.7 %)	88	(65.7 %)	
Anti-PR3 (U/mL)	0.2	(0.2–0.2)	2.4	(0.2–74.3)	0.2	(0.2–0.3)	<0.001^†#^
Anti-MPO (U/mL)	0.2	(0.2–0.2)	0.2	(0.2–0.2)	32.0	(8.9–89.5)	<0.001^‡#^
ANA result							
Positive (≥1:160)	49	(30.6 %)	8	(33.3 %)	67	(55.8 %)	<0.001**
ANA titer	160.0	(80.0–320.0)	160.0	(80.0–320.0)	160.0	(100.0–640.0)	0.067
RBC ( × 10^6^/μL)	4.4	(4.0–4.8)	3.8	(3.2–4.6)	3.5	(2.8–4.2)	<0.001^†‡^
HGB (g/dL)	13.3	(12.0–14.7)	11.5	(9.6–13.5)	10.4	(8.5–12.5)	<0.001^†‡^
Platelet ( × 10^3^/μL)	245.0	(202.3–294.8)	244.5	(182.8–330.3)	222.0	(171.0–303.5)	0.198
WBC (μL)	7050.0	(5930.0–9235.0)	5850.0	(4445.0–9767.5)	7190.0	(5540.0–10145.0)	0.174
Neutrophil count (μL)	4548.2	(3511.2–6575.6)	4819.115	(2705.8–7841.4)	5443.5	(3557.8–8587.5)	0.044*
Creatinine (mg/dL)	0.8	(0.7–1.0)	0.8	(0.6–1.2)	1.6	(0.8–5.2)	<0.001^‡#^
CRP (mg/dL)	0.2	(0.0–0.8)	0.2	(0.1–2.6)	0.2	(0.1–1.0)	0.807
UPCR	53.1	(0.2–319.2)	109.7	(3.7–1836.4)	322.6	(3.8–2950.1)	0.007**
Comorbidities							
Hypertension	40	(23.5 %)	7	(23.3 %)	54	(40.3 %)	0.005^‡^
DM	32	(18.8 %)	3	(10.0 %)	22	(16.4 %)	0.480
Hyperlipidemia	36	(21.2 %)	7	(23.3 %)	25	(18.7 %)	0.789
CKD	23	(13.5 %)	11	(36.7 %)	82	(61.2 %)	<0.001^†‡^
COPD	10	(5.9 %)	2	(6.7 %)	11	(8.2 %)	0.728
ILD	7	(4.1 %)	2	(6.7 %)	10	(7.5 %)	0.445
TB	4	(2.4 %)	0	(0.0 %)	4	(3.0 %)	0.626
AAV	3	(1.8 %)	10	(33.3 %)	62	(46.3 %)	<0.001^†‡^
MPA	3	(1.8 %)	1	(3.3 %)	56	(41.8 %)	<0.001^‡#^
GPA	0	(0.0 %)	9	(30.0 %)	2	(1.5 %)	<0.001^†#^
EGPA	0	(0.0 %)	0	(0.0 %)	4	(3.0 %)	0.049*
RA	17	(10.0 %)	1	(3.3 %)	5	(3.7 %)	0.073
SLE	10	(5.9 %)	10	(33.3 %)	25	(18.7 %)	<0.001^†‡^
SSc	3	(1.8 %)	0	(0.0 %)	3	(2.2 %)	0.705
UC	11	(6.5 %)	0	(0.0 %)	2	(1.5 %)	0.043*
Medications							
Glucocorticoid	35	(20.6 %)	9	(30.0 %)	24	(17.9 %)	0.329
Azathioprine	31	(18.2 %)	7	(23.3 %)	43	(32.1 %)	0.020^‡^
Cyclophosphamide	5	(2.9 %)	9	(30.0 %)	27	(20.1 %)	<0.001^†‡^
MTX	12	(7.1 %)	4	(13.3 %)	3	(2.2 %)	0.033*
Rituximab	0	(0.0 %)	6	(20.0 %)	16	(11.9 %)	<0.001^†#^

Data are expressed as n (%) or median (interquartile range [IQR]); Statistical analysis by the chi-square test or the Kruskal-Wallis test. *p < 0.05, **p < 0.01. ^†^Atypical ANCA vs. c-ANCA; ^‡^ Atypical ANCA vs. *p*-ANCA; ^#^c-ANCA vs. *p*-ANCA. ANA: anti-nuclear cell antibody; UPCR: urine protein/creatinine ratio; DM: diabetes mellitus; CKD: chronic kidney syndrome; COPD: chronic obstructive pulmonary disease; ILD: interstitial lung disease; TB: tuberculosis; AAV: ANCA-associated vasculitis; MPA: microscopic polyangiitis; GPA: Granulomatosis with polyangiitis; EGPA: eosinophilic granulomatosis with polyangiitis; RA: rheumatoid arthritis; SLE: systemic lupus erythematosus; SSc: systemic sclerosis; UC: ulcerative colitis; MTX: methotrexate.

### 3.2 Factors associated with a-ANCA

In Table 2, the results of a logistic regression performed to identify variables and diseases associated with a-ANCA have been reported. Compared with the typical ANCA group, participants in the a-ANCA group exhibited a higher levels of RBC, HGB, a lower levels of anti-MPO, serum creatinine, a lower chance of hypertension, CKD, AAV (OR: 0.02, 95 % CI: 0.01–0.07, *p* < 0.001), and SLE (OR: 0.23, 95 % CI: 0.11–0.48, *p* < 0.001), but a higher association with RA (OR: 2.99, 95 % CI: 1.15–7.83, *p* = 0.025) and UC (OR: 5.50, 95 % CI: 1.20–25.29, *p* = 0.028).

**Table 2:** Age and sex-adjusted factors associated with atypical ANCA.

	Univariate				Multivariable†			
	OR	95%CI		*p* value	OR	95%CI		*p* value
Age	1.00	(0.99-	1.01)	0.952				
Sex								
Female	Reference							
Male	1.18	(0.76-	1.83)	0.460				
Anti-PR3 (U/mL)	0.87	(0.54-	1.40)	0.575	0.87	(0.55-	1.39)	0.564
Anti-MPO (U/mL)	0.32	(0.12-	0.83)	0.019*	0.33	(0.13-	0.83)	0.019*
ANA result								
Negative (<1:160)	Reference				Reference			
Positive (≥1:160)	0.41	(0.25-	0.65)	<0.001**	0.40	(0.25-	0.65)	<0.001**
ANA titer	1.00	(1.00-	1.00)	0.055	1.00	(1.00-	1.00)	0.054
RBC ( × 10^6^/μL)	2.95	(2.15-	4.05)	<0.001**	3.64	(2.54-	5.21)	<0.001**
HGB (g/dL)	1.53	(1.37-	1.71)	<0.001**	1.59	(1.41-	1.79)	<0.001**
Platelet ( × 10^3^/μL)	1.00	(1.00-	1.00)	0.197	1.00	(1.00-	1.00)	0.214
WBC (μL)	1.00	(1.00-	1.00)	0.123	1.00	(1.00-	1.00)	0.090
Neutrophil count(μL)	1.00	(1.00-	1.00)	0.004**	1.00	(1.00-	1.00)	0.002**
Creatinine (mg/dL)	0.65	(0.55-	0.77)	<0.001**	0.62	(0.52-	0.75)	<0.001**
UPCR	1.00	(1.00-	1.00)	0.120	1.00	(1.00-	1.00)	0.095
Comorbidities								
Hypertension	0.52	(0.32-	0.84)	0.007**	0.49	(0.30-	0.81)	0.005**
DM	1.29	(0.73-	2.29)	0.385	1.33	(0.73-	2.42)	0.347
Hyperlipidemia	1.11	(0.65-	1.89)	0.706	1.11	(0.65-	1.92)	0.695
CKD	0.12	(0.07-	0.20)	<0.001**	0.10	(0.06-	0.18)	<0.001**
COPD	0.73	(0.31-	1.71)	0.462	0.65	(0.27-	1.61)	0.354
ILD	0.54	(0.21-	1.42)	0.213	0.52	(0.20-	1.38)	0.190
TB	0.96	(0.24-	3.92)	0.959	0.92	(0.22-	3.82)	0.908
AAV	0.02	(0.01-	0.07)	<0.001**	0.02	(0.01-	0.07)	<0.001**
RA	2.93	(1.12-	7.62)	0.028*	2.99	(1.15-	7.83)	0.025*
SLE	0.23	(0.11-	0.48)	<0.001**	0.23	(0.11-	0.48)	<0.001**
SSc	0.96	(0.19-	4.85)	0.965	0.93	(0.18-	4.69)	0.926
UC	5.60	(1.22-	25.68)	0.027*	5.50	(1.20-	25.29)	0.028*

OR: odds ratio; CI: confidence interval. Statistical analysis by logistic regression, *p < 0.05, **p < 0.01. ANA: anti-nuclear cell antibody; UPCR: urine protein/creatinine ratio; DM: diabetes mellitus; CKD: chronic kidney syndrome; COPD: chronic obstructive pulmonary disease; ILD: interstitial lung disease; TB: tuberculosis; AAV: ANCA-associated vasculitis; RA: rheumatoid arthritis; SLE: systemic lupus erythematosus; SSc: systemic sclerosis; UC: ulcerative colitis.

### 3.3 Risks associated with renal failure

**Fig. 1:**
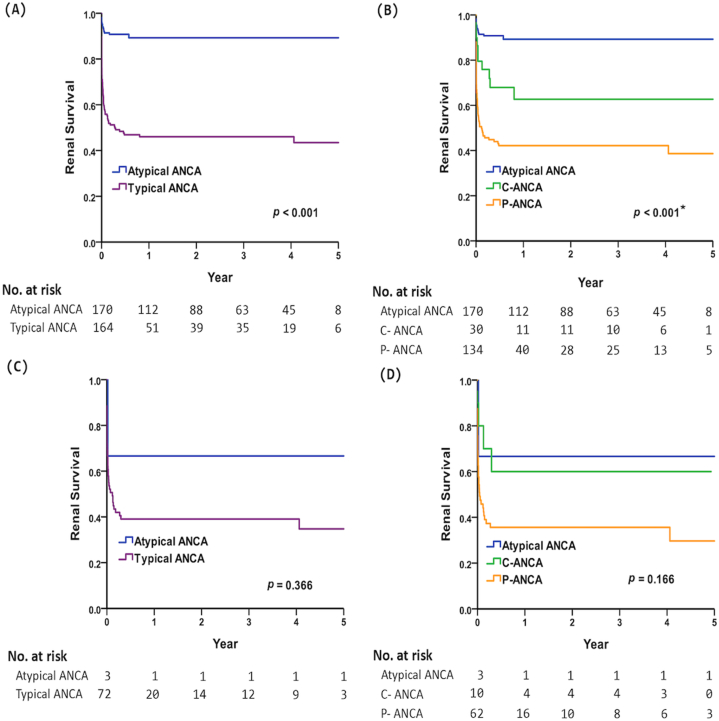
Kaplan–Meier curve depicting 5-year renal survival in all patients with (A) atypical ANCA vs. typical ANCA, (B) atypical ANCA vs. c-ANCA and *p*-ANCA; AAV patients with (C) atypical ANCA vs. typical ANCA, and (D) atypical ANCA vs. c-ANCA and *p*-ANCA. *Post hoc analysis by the log-rank test, all pairwise *p* < 0.05.

Using Cox regression analysis, we found that higher levels of anti-MPO and creatinine, and hypertension, and AAV were independent factors associated with renal survival in patients with a-ANCA. Moreover, patients with a-ANCA used fewer medications such as cyclophosphamide and rituximab compared to patients with typical ANCA group (Table 3). Additionally, patients with a-ANCA exhibited the highest renal survival rates as compared with the typical ANCA group (Fig. 1A). The 5-year renal survival was 89.3 % and 43.5 % in all patients with a-ANCA and typical ANCA, respectively (*p* < 0.001 by Kaplan–Meier analysis). As shown in Fig. 1B, patients with a-ANCA had the best renal survival rate (89.3 %; *p* < 0.001 by Kaplan–Meier analysis) compared with those in the c-ANCA (62.7 %) and *p*-ANCA (38.6 %) groups. Furthermore, AAV patients with a-ANCA had a numerically higher 5-year renal survival rate as compared with the typical ANCA group (66.7 % vs. 34.7 %, *p* = 0.366, Fig. 1C). AAV patients with a-ANCA tended to have a better, but not statistically significant renal survival rate compared with those in the c-ANCA and *p*-ANCA (66.7 % vs. 60.0 % vs. 29.7 %, *p* = 0.166, Fig. 1D).

**Table 3:** Age and sex-adjusted factors associated with renal survival and mortality.

	Factors associated with renal survival				Factors associated with mortality			
	Univariate		Age and sex-adjusted		Univariate		Age and sex-adjusted	
	HR (95 % CI)	*p*-value	HR (95 % CI)	*p*-value	HR (95 % CI)	*p*-value	HR (95 % CI)	*p*-value
Age	1.02(1.01–1.03)	0.001**			1.07(1.05–1.10)	<0.001**		
Sex								
Female	Reference				Reference			
Male	1.17(0.79–1.73)	0.431			1.38(0.76–2.50)	0.285		
Group								
Typical ANCA	Reference		Reference		Reference		Reference	
Atypical ANCA	0.15(0.09–0.25)	<0.001**	0.14(0.08–0.24)	<0.001**	0.34(0.18–0.67)	0.002**	0.31(0.16–0.60)	0.001**
Anti-PR3 (U/mL)	1.00(0.98–1.01)	0.589	1.00(0.98–1.01)	0.806	0.96(0.85–1.09)	0.536	0.98(0.88–1.08)	0.652
Anti-MPO (U/mL)	1.01(1.01–1.02)	<0.001**	1.01(1.01–1.02)	<0.001**	1.01(1.01–1.02)	0.001**	1.01(1.00–1.02)	0.012*
ANA result								
Negative (<1:160)	Reference		Reference		Reference			
Positive (≥1:160)	1.37(0.91–2.07)	0.133	1.21(0.80–1.84)	0.370	1.45(0.76–2.73)	0.257	1.11(0.58–2.10)	0.756
ANA titer	1.00(1.00–1.00)	0.934	1.00(1.00–1.00)	0.973	1.00(1.00–1.00)	0.213	1.00(1.00–1.00)	0.242
RBC ( × 10^6^/μL)	0.38(0.30–0.47)	<0.001**	0.39(0.31–0.49)	<0.001**	0.35(0.25–0.49)	<0.001**	0.43(0.30–0.61)	<0.001**
HGB (g/dL)	0.70(0.66–0.76)	<0.001**	0.71(0.66–0.76)	<0.001**	0.69(0.62–0.77)	<0.001**	0.70(0.62–0.79)	<0.001**
Platelet ( × 10^3^/μL)	0.99(0.99–1.00)	<0.001**	1.00(0.99–1.00)	<0.001**	1.00(0.99–1.00)	0.107	1.00(1.00–1.00)	0.378
WBC (μL)	1.00(1.00–1.00)	0.305	1.00(1.00)1.00	0.484	1.00(1.00–1.00)	<0.001**	1.00(1.00–1.00)	<0.001**
Neutrophil count(μL)	1.00(1.00–1.00)	0.015*	1.00(1.00–1.00)	0.067	1.00(1.00–1.00)	<0.001**	1.00(1.00–1.00)	<0.001**
Creatinine (mg/dL)	1.38(1.32–1.45)	<0.001**	1.39(1.32–1.46)	<0.001**	1.13(1.04–1.22)	0.006**	1.13(1.03–1.24)	0.010*
CRP (mg/dL)	1.04(0.97–1.13)	0.262	1.05(0.97–1.15)	0.218	0.97(0.70–1.34)	0.839	0.96(0.65–1.43)	0.846
UPCR	1.00(1.00–1.00)	<0.001**	1.00(1.00–1.00)	<0.001**	1.00(1.00–1.00)	0.768	1.00(1.00–1.00)	0.608
Comorbidities								
Hypertension	3.69(2.50–5.46)	<0.001**	3.33(2.22–5.02)	<0.001**	1.35(0.73–2.50)	0.335	0.71(0.38–1.35)	0.301
DM	1.41(0.89–2.24)	0.145	1.13(0.70–1.82)	0.625	1.71(0.86–3.39)	0.123	1.01(0.50–2.01)	0.985
Hyperlipidemia	1.67(1.09–2.57)	0.019*	1.46(0.94–2.25)	0.092	1.16(0.57–2.35)	0.683	0.74(0.37–1.52)	0.415
CKD	–		–		3.48(1.88–6.43)	<0.001**	2.31(1.23–4.33)	0.009**
COPD	1.17(0.57–2.40)	0.675	0.79(0.37–1.70)	0.549	2.89(1.28–6.49)	0.010*	1.11(0.45–2.73)	0.814
ILD	0.51(0.16–1.61)	0.252	0.43(0.14–1.37)	0.153	1.41(0.44–4.56)	0.565	1.19(0.37–3.85)	0.773
TB	1.86(0.68–5.05)	0.225	1.29(0.46–3.59)	0.632	0.90(0.12–6.52)	0.914	0.35(0.05–2.59)	0.301
AAV	3.29(2.23–4.87)	<0.001**	3.11(2.10–4.61)	<0.001**	1.10(0.56–2.18)	0.780	0.91(0.46–1.80)	0.789
RA	0.78(0.34–1.77)	0.545	0.70(0.31–1.60)	0.394	0.61(0.15–2.50)	0.488	0.40(0.10–1.68)	0.212
SLE	0.89(0.50–1.59)	0.684	1.03(0.57–1.86)	0.922	0.39(0.12–1.27)	0.119	0.55(0.17–1.80)	0.326
SSc	1.19(0.29–4.83)	0.807	1.04(0.26–4.23)	0.957	1.89(0.26–13.75)	0.531	1.53(0.21–11.23)	0.674
UC	0.05(0.00–2.53)	0.132	–		0.05(0.00–22.93)	0.332	–	
Medication								
Glucocorticoid	0.81(0.49–1.34)	0.414	0.78(0.47–1.29)	0.334	0.33(0.12–0.93)	0.036*	0.30(0.11–0.85)	0.023*
Azathioprine	0.70(0.43–1.13)	0.145	0.79(0.48–1.29)	0.341	0.36(0.14–0.92)	0.034*	0.49(0.19–1.26)	0.139
Cyclophosphamide	2.05(1.28–3.28)	0.003**	2.34(1.44–3.79)	0.001**	0.66(0.24–1.85)	0.433	0.93(0.33–2.67)	0.899
MTX	0.29(0.07–1.17)	0.081	0.33(0.08–1.33)	0.120	0.36(0.05–2.62)	0.314	0.61(0.08–4.50)	0.630
Rituximab	2.20(1.23–3.94)	0.008**	2.63(1.46–4.73)	0.001**	0.98(0.30–3.18)	0.978	1.64(0.50–5.34)	0.415

HR: hazard ratio; CI: confidence interval. Statistical analysis by Cox proportional hazard regression. *p < 0.05, **p < 0.01. ANA: anti-nuclear cell antibody; UPCR: urine protein/creatinine ratio; DM: diabetes mellitus; CKD: chronic kidney syndrome; COPD: chronic obstructive pulmonary disease; ILD: interstitial lung disease; TB: tuberculosis; AAV: ANCA-associated vasculitis; RA: rheumatoid arthritis; SLE: systemic lupus erythematosus; SSc: systemic sclerosis; UC: ulcerative colitis; MTX: methotrexate.

### 3.4 Risks associated with patient mortality

**Fig. 2:**
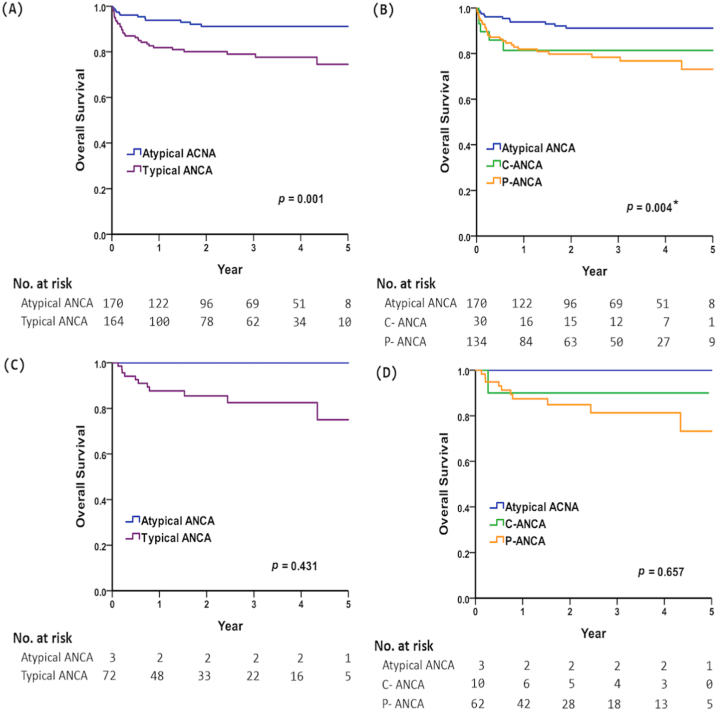
Kaplan–Meier curve depicting 5-year overall survival in all patients with (A) atypical ANCA vs. typical ANCA, (B) atypical ANCA vs. c-ANCA and *p*-ANCA; AAV patients with (C) atypical ANCA vs. typical ANCA, and (D) atypical ANCA vs. c-ANCA and *p*-ANCA. *Post hoc analysis by log-rank test, atypical ANCA vs. *p*-ANCA, *p* < 0.01.

Participants in the typical ANCA group had a higher 5-year mortality rate than those in the a-ANCA group (25.5 % vs. 8.8 %, *p* = 0.001, Fig. 2A). Moreover, patients with *p*-ANCA had the highest mortality rate, compared with those in the c-ANCA and a-ANCA groups (26.9 % vs. 18.6 % vs. 8.8 %, *p* = 0.004, Fig. 2B). Additionally, AAV patients with typical ANCA had a numerically higher 5-year mortality rate than a-ANCA group (25.0 % vs. 0 %, *p* = 0.431, Fig. 2C); AAV patients with *p*-ANCA also had a numerically higher mortality rate than c-ANCA and a-ANCA groups (26.8 % vs. 10 % vs. 0 %, *p* = 0.657, Fig. 2D). Finally, a-ANCA positivity, a lower of anti-MPO, serum creatinine levels, and absence of CKD were associated with a lower mortality rate (Table 3).

## 4 Discussion

This real-world, hospital-based study demonstrated that, compared with patients who had typical ANCA, patients with a-ANCA had a higher correlation with RA and UC and exhibited better prognosis in terms of renal survival and overall survival after adjustment for age and sex.

The proportion of a-ANCA positivity is rare in patients with AAV. Jan D. et al. analyzed 866 sera from controls without ANCA-associated vasculitis and found that only 5 (0.58 %) were tested positive for a-ANCA; none of sera from 251 patients with ANCA-associated vasculitis were tested positive for a-ANCA [30]. Previous studies of a-ANCA mainly enrolled patient population of one autoimmune disease [6,[8], [9], [10], [11], [12], [13], [14], [15]]. However, our study cohort involved a mixed population who were tested for ANCA in tertiary referral hospital by a wide range of physicians in different subspecialties. We believed that our study design contributed to a similar proportion of typical ANCA and a-ANCA positivity in our study.

According to numerous reports, 37–83 % of individuals with a-ANCA were found to have UC [[7], [8], [9], [10], [11], [12], [13]]. These findings align with our study, supporting a significant association between participants with a-ANCA and UC. Furthermore, we discovered the patients with a-ANCA had a higher association with RA, which was not previously reported in the literature. A-ANCA, differing from typical ANCA in antigen specificity, may contribute to the pathogenesis of UC and RA through several plausible mechanisms. Firstly, a-ANCA plays a role in UC and RA by triggering an abnormal immune response. In contrast to typical ANCA, it targets different cytoplasmic constituents, potentially including bacterial permeases or human lysosome-associated proteins. This results in a unique autoimmune response, leading to increased endothelial cell activation and neutrophil extracellular trap (NET) formation, thereby exacerbating inflammation in UC and RA. Furthermore, the impact of a-ANCA varies between RA and SLE due to distinct autoimmune pathways. In RA, it primarily induces synovial inflammation and joint damage, potentially intensified by a-ANCA-related neutrophil activation and endothelial damage. Conversely, in SLE, the primary mechanism involves immune complex formation and complement activation. Due to the unique antigenic targets of a-ANCA, its influence on SLE may be limited, resulting in a comparatively lower incidence in our cohort. The incidence of a-ANCA and typical ANCA has not been compared in a cohort tested for ANCA by indirect immunofluorescence assay [30]. In clinical practice, when managing patients with a-ANCA, physicians should assess for signs of RA and UC to formulate a timely therapeutic plan for these patients.

A previous study showed that 25.5 % of ANCA-positive patients may progress to renal failure and would need hemodialysis [23]. We found that renal survival was superior in the a-ANCA group compared with the typical ANCA group. Furthermore, we analyzed the renal survival between the c-ANCA and *p*-ANCA groups and found a higher renal survival in patients with c-ANCA than in those with *p*-ANCA. In our study, the 5-year renal survival was 89.3 %, 62.7 %, and 38.6 % in patients with a-ANCA, c-ANCA, and *p*-ANCA, respectively. Compared with the typical ANCA group, patients with a-ANCA had better renal outcome. However, we believe that unidentified contributing factors for deterioration of renal function may still exist in patients with a-ANCA. Furthermore, our result demonstrated that higher levels of anti-MPO, creatinine, and combined with hypertension and AAV were concomitant risks for renal injury in patients with a-ANCA. Although patients with a-ANCA had better renal survival, it is possible that renal replacement may be needed in a small (10.7 %) proportion of these patients. The cause of kidney failure that required renal replacement therapy in 17 patients with a-ANCA included DM nephropathy (n = 5, 29.4 %); hypertensive heart disease, HCVD (n = 5, 29.4 %); SLE (n = 1, 5.9 %); focal segmental glomerulosclerosis, FSGS (n = 1, 5.9 %); renal cell carcinoma, RCC (n = 1, 5.9 %); urothelial carcinoma (n = 1, 5.9 %), and unknown (n = 3, 17.7 %). While our study did not establish a direct association between a-ANCA and renal outcomes, the data from the age and sex-adjusted model suggested a potential correlation between a-ANCA and renal survival. Despite the generally favorable prognosis associated with a-ANCA compared to typical ANCA, it is noteworthy that some individuals still encounter adverse renal outcomes. Future studies are warranted to elucidate the relationship between a-ANCA and the risk of kidney failure.

In our study, we found that mortality was higher in the typical ANCA group as compared to the a-ANCA group. Our study is the first to investigate the long-term mortality rate among patients with typical ANCA and a-ANCA. The 5-year mortality rate was worse in patients with *p*-ANCA, followed by that in patients with c-ANCA and, finally, a-ANCA. We identified that CKD were correlated with mortality in patients with a-ANCA. Higher levels of anti-MPO and serum creatinine were associated with higher mortality risks in participants with a-ANCA. In our study, the mean age of death was 85 years in the a-ANCA group, which has never been reported in previous studies. According to the Ministry of Health and Welfare, Taiwan, the life expectancy of the Taiwanese individuals at birth was 81.3 years in 2020 [31]. In comparison to the general population, we observed that the average lifespan of patients with a-ANCA was not significantly affected. This suggests that typical ANCA associated with AAV represents a spectrum of the disease, ranging from isolated organ involvement to potentially life-threatening fulminant disease, as opposed to a-ANCA [22]. The milder clinical course linked to a-ANCA is influenced by its unique relationship with typical ANCA and AAV, a group of autoimmune diseases often presenting as small-vessel vasculitis. A-ANCA's distinctive antigenic specificity contributes to a less aggressive disease phenotype, potentially sparing critical organs like the kidneys and lungs. Variations in pathway activation between typical ANCA and a-ANCA influence tissue damage extent and nature, impacting overall disease severity. The milder outcomes associated with a-ANCA may result from a blend of antigenic specificity, immunopathogenic pathways, and genetic/environmental influences. Our study further revealed that among the 12 patients with a-ANCA who passed away, the causes included cancer (n = 2; 16.7 %), cardiovascular disease (n = 2; 16.7 %), infection (n = 2; 16.7 %), and other causes (n = 6; 50.0 %; specifically pulmonary disease, connective tissue disease, natural death, etc.) (data not shown). It is imperative for rheumatologists to diligently monitor for associated conditions such as UC and RA and provide necessary interventions to mitigate risks and prevent undesirable outcomes.

There were several limitations in our study. First, our study design lacked an ANCA-negative group as the negative control, thereby hindering our ability to be entirely certain whether patients with a-ANCA exhibited a worse renal outcome and patient mortality compared to the general population. Additionally, the study enrolled individuals who visited hospitals due to specific signs and symptoms necessitating ANCA testing, rather than drawing from the general population or individuals with autoimmune rheumatic diseases. However, our study is the first to demonstrate that the overall renal survival and mortality for patients with a-ANCA could be eventful and should not be neglected. Second, due to the retrospective design, the possibility of missing data could not be avoided. The median follow-up time was only 2.2 years, which might not be long enough to observe renal outcome and mortality. In addition, ANCA tests were performed to facilitate the diagnosis of AAV at the physicians’ discretion. The indication of ANCA testing for every enrolled participant was not prospectively collected. Indication bias may play an important factor in this study (i.e., ANCA may be tested in very ill patients or in presence of organ failure/dysfunction which can overestimate the association between ANCA test result and outcome). However, our study truly reflects the daily practice of clinicians in the real-world setting. Third, the study population comprised only hospital-based Taiwanese individuals. Therefore, our results might not be generalizable to another ethnic group or community-based patient group.

In conclusion, patients with a-ANCA might have a distinct prognosis than patients with typical ANCA. Although the clinical outcome of a-ANCA -positive patients was better, it was not uneventful. Clinicians should be aware of the concomitant comorbidities associated with a-ANCA and proactively implement strategies to improve the clinical outcome in this group of patients.

## Declarations

### Ethics statement

This study was approved (CE22213A) by the Institutional Review Board of Taichung Veterans General Hospital, Taiwan.

## Funding statement

This study was funded by National Science and Technology Council [grant numbers, NSTC -111-2634-F-A49-014, NSTC-111-2218-E−039-001, and NSTC-111-2314-B-005-007-MY3], and 10.13039/501100010101Taichung Veterans General Hospital, Taiwan [grant numbers TCVGH-1127301C, TCVGH-1127302D, and TCVGH-YM1120110].

## Data availability statement

All data used in this study in this article are available upon reasonable request to the corresponding author.

## CRediT authorship contribution statement

**Chiao-Chi Ou:** Writing - review & editing, Writing - original draft, Methodology, Conceptualization. **Yen-Ching Wu:** Writing - review & editing, Methodology. **Jun-Peng Chen:** Writing - review & editing, Methodology, Formal analysis, Data curation. **Wen-Nan Huang:** Writing - review & editing, Supervision, Resources. **Yi-Hsing Chen:** Writing - review & editing, Supervision, Resources, Methodology. **Yi-Ming Chen:** Writing - review & editing, Writing - original draft, Resources, Methodology, Funding acquisition, Data curation, Conceptualization.

## Declaration of generative AI and AI-assisted technologies in the writing process

During the preparation of this work the authors used ChatGPT in order to improve language and readability. After using this tool/service, the authors reviewed and edited the content as needed and take full responsibility for the content of the publication.

## Declaration of competing interest

The authors declare that they have no known competing financial interests or personal relationships that could have appeared to influence the work reported in this paper.
