# Supplementary material for: Association of atypical anti-neutrophil cytoplasmic antibody with comorbidities and outcome in a hospital-based population
Source: Heliyon. 2024 Jan 6;10(1):e24105. doi: 10.1016/j.heliyon.2024.e24105 (PMC10792567; doi:10.1016/j.heliyon.2024.e24105)
Supplement: Multimedia component 1 [file mmc1.docx]

**Supplementary files:**

**Supplement Table 1.** ICD-9-CM and ICD-10-CM codes for comorbidity identification.

| Comorbidities | ICD-9-CM code | ICD-10-CM code |
| --- | --- | --- |
| Hypertension | 401 | I10 |
| Diabetes mellitus (DM) | 249.X | E08.X |
| Hyperlipidemia | 272.x | E78.X |
| Chronic kidney disease (CKD) | 580.X, 583.4, 585.X, 586 | N00.X, N05.9, N18.X, N19 |
| Chronic obstructive pulmonary disease (COPD) | 496 | J44.X |
| Interstitial lung disease (ILD) | 421.X | J84.X |
| Tuberculosis (TB) | 010-018 | A15-A19 |
| ANCA-associated vasculitis (AAV) | 446.X, 447.X | M30.X, I77.X |
| Rheumatoid arthritis (RA) | 714.0 | M06.9 |
| Systemic lupus erythematosus (SLE) | 710.0 | M32.10 |
| Systemic sclerosis (SSc) | 710.1 | M34.0 |
| Ulcerative colitis (UC) | 556.X | K51.X |

ICD-9-CM: International Classification of Diseases, Ninth Revision, Clinical Modification, ICD-10-CM: International Classification of Diseases, Tenth Revision, Clinical Modification.
